# ESM‐scan—A tool to guide amino acid substitutions

**DOI:** 10.1002/pro.5221

**Published:** 2024-11-20

**Authors:** Massimo G. Totaro, Uršula Vide, Regina Zausinger, Andreas Winkler, Gustav Oberdorfer

**Affiliations:** ^1^ Institute of Biochemistry Graz University of Technology Graz Austria; ^2^ BioTechMed Graz Austria

**Keywords:** in‐silico deep mutational scanning, protein design, protein engineering, protein function, structural biology

## Abstract

Protein structure prediction and (re)design have gone through a revolution in the last 3 years. The tremendous progress in these fields has been almost exclusively driven by readily available machine learning algorithms applied to protein folding and sequence design problems. Despite these advancements, predicting site‐specific mutational effects on protein stability and function remains an unsolved problem. This is a persistent challenge, mainly because the free energy of large systems is very difficult to compute with absolute accuracy and subtle changes to protein structures are hard to capture with computational models. Here, we describe the implementation and use of ESM‐Scan, which uses the ESM zero‐shot predictor to scan entire protein sequences for preferential amino acid changes, thus enabling in silico deep mutational scanning experiments. We benchmark ESM‐Scan on its predictive capabilities for stability and functionality of sequence changes using three publicly available datasets and proceed by experimentally testing the tool's performance on a challenging test case of a blue‐light‐activated diguanylate cyclase from *Methylotenera* species (*Ms*LadC), where it accurately predicted the importance of a highly conserved residue in a region involved in allosteric product inhibition. Our experimental results show that the ESM‐zero shot model is capable of inferring the effects of a set of amino acid substitutions in their correlation between predicted fitness and experimental results. ESM‐Scan is publicly available at https://huggingface.co/spaces/thaidaev/zsp.

## 1 INTRODUCTION

Proteins play a central role in various aspects of life, operating as fine‐tuned molecular assemblies. These systems’ properties define structure and dynamics so that even single alterations in the amino acid sequence can profoundly affect their functionality (Casadio et al., 2011; Tokuriki & Tawfik, 2009; Zhang et al., 2018). Determining the amino acid substitution effects on protein structure, stability and function remain challenging despite continuous advances in structural and functional analyses (Fersht, 1998; Gromiha, 2007; Shoichet et al., 1995). Experimental approaches are often time‐consuming and costly, even with high‐throughput screening methods.

The earliest computational tools developed for this task: Eris, FoldX, and Rosetta‐ddG to name a few, leverage a combination of energy calculations, evolutionary insights, and statistical analyses (Hopf et al., 2017; Kellogg et al., 2010; Ng & Henikoff, 2003; Schymkowitz et al., 2005; Smith & Kortemme, 2011; Yin et al., 2007; Yue et al., 2005). While achieving moderate success rates, these tools require substantial additional knowledge of the methods and the systems on the user's part (Broom et al., 2017; Hsu et al., 2022; Schmitz et al., 2021; Yamaguchi & Saito, 2021). Early machine learning approaches demonstrated good predictive power; however, they tended to overfit training data, limiting their ability to generalize to proteins beyond the original dataset (Avery et al., 2022; Bradford & Westhead, 2004; Rao et al., 2019; Verkuil et al., 2022). Expanding beyond these early approaches, deep learning models harness vast sequence datasets and experimental information, autonomously learning from them unsupervised (Gilmer et al., 2017; Riesselman et al., 2018). These models better capture the individual amino acids' intricate and nonlinear contributions to protein stability and functionality (Ingraham et al., 2019; Sanderson et al., 2023; Mansoor et al., 2023). However, the generalization capacity of deep learning models remains an open topic, with persistent concerns about the risk of overfitting and the inconsistent transfer of learned knowledge to novel proteins beyond the training dataset (Lin et al., 2023; Notin, 2022).

Arguably, the most well‐known example of a deep learning model in biosciences is AlphaFold (AF) and its successive improvements (AF2 and AF3), widely acknowledged as a transformative breakthrough in computational protein structural biology (Abramson et al., 2024; Jumper et al., 2021; Senior et al., 2020; Tunyasuvunakool et al., 2021). Tweaks in the original architecture endowed the model with versatility, enabling it to undertake different tasks such as predicting multimeric assemblies, exploring alternative conformations, and evaluating the impact of single amino acid substitutions on protein stability and function with varying levels of accuracy (Bryant et al., 2022; McBride et al., 2022; Pak et al., 2023; Sala et al., 2023; Wayment‐Steele et al., 2023). Further developments, like the AlphaMissense model by Cheng et al. (2023), generated a database where single‐point variants of the human proteome can be queried for fitness. Another promising research area in deep learning explores language models. These models learn natural language syntax, semantics, and contextual nuances and can generate new meaningful sentences. Drawing an analogy, protein sequences are akin to natural language texts, where amino acids correspond to letters, secondary structures to words, complete sequences to sentences, and assemblies of several proteins to paragraphs (Ferruz et al., 2022; Lin et al., 2023; Wenzel, 2023; Z. Zhang, 2023). Protein‐specific language models like ESM, BERT, and their derivatives (Brandes, Ofer, et al., 2022; Chowdhury et al., 2022; Rives et al., 2021), based on the transformer architecture (Vaswani et al., 2017), are capable of learning and extrapolating hidden protein patterns that elude traditional energy‐based methods (Chandra et al., 2023). An interesting aspect of these models is their potential use as zero‐shot learners, predictors that can run on sets of classes not included in the training data without additional training (Lampert et al., 2009; Larochelle et al., 2008).

The outlined modeling approaches can potentially transform day‐to‐day research for professionals working in biosciences. However, while some progress has been made, setting up and deploying an ML model is still nontrivial for most users. Moreover, data availability and curation are fundamental in the training and testing phases. Finally, the absence of a computational gold standard method currently hinders the direct transferability of model performances to varied use cases (Avery et al., 2022).

Here, we introduce ESM‐Scan, a computational tool leveraging language models from the ESM family to rapidly and efficiently infer the fitness of amino acid substitutions on a given sequence (Brandes, Goldman, et al., 2022; Meier et al., 2021). Our tool is publicly available and requires no set‐up from the user, ensuring ease of use. More skilled users can download the source code directly and adapt the tool for more specialized use cases. To illustrate the tool's practical performance, expectations, and limitations, we benchmark its performance against deep mutational scanning datasets also used to benchmark other computational tools described in the literature.

We further provide a demonstration case of the tool using the blue‐light‐activated diguanylate cyclase from *Methylotenera species* (*Ms*LadC), a well‐characterized in‐house model system (Vide et al., 2023). *Ms*LadC acts as a light‐regulatable switch alternating between two distinct dimeric conformations: a compact inactive state and an extended active state. In addition to the light sensitivity of the flavin‐binding LOV domain and the enzymatic activity of the GGDEF domain, the latter also features a conserved allosteric site for noncompetitive product inhibition (Schirmer, 2016). In *Ms*LadC, this site is also an integral part of an extensive inhibitory interface between sensor and effector domains, governing the protein's inactive, dark state (Vide et al., 2023). Moreover, in other GGDEF domains, this region has been observed to be allosterically coupled to the active site and may also play a role in regulatory protein–protein interactions (Dahlstrom et al., 2016; Hengge, 2021). Originally, we attempted to mitigate the allosteric product inhibition using amino acid substitutions suggested in the literature on related proteins (Teixeira et al., 2021), aiming at increasing the abundance of elongated active conformations for their biophysical characterization. However, these initial variants resulted in low expression yields or nonfunctional proteins. With ESM‐Scan, using only the amino acid sequence as input, we could obtain promising alternative single amino acid replacements, less detrimental to protein fitness, allowing variant characterization. We find that ESM‐scan scores correlate with functional observations relating to different properties including solubility, cofactor loading, and enzymatic activity, which, coupled with stability, flexibility, and structural integrity, determine said fitness.

Overall, ESM‐Scan offers a minimal‐overhead and user‐friendly resource to the scientific community, guiding the selection of possible amino acid substitutions by predicting their effects and empowering further specialized applications.

## 2 METHODS

### 2.1 ESM‐scan

Our ESM‐Scan tool uses protein language models of the ESM family, pre‐trained for masked token inference (Devlin, 2018). In masked language model inferences, each residue is assigned a probability score, based on its sequence context. Notably, a single residue alteration in an otherwise identical context produces a distinct score that estimates the impact of such substitution. The inference values range marks the transition between enhanced (markedly positive values), neutral (mildly negative to positive), and reduced (more negative) fitness for the tested variant. The latest iteration of ESM‐Scan is hosted on HuggingFace (huggingface.co/spaces/thaidaev/zsp), featuring a user‐friendly web interface that facilitates model inferences with minimal user configuration and provides directly interpretable results.

### 2.2 Benchmarking datasets

The benchmarking datasets cover various use cases and originate from different experimental setups. For energy calculations, the Rosetta software suite stands as one of the industry standards (Das & Baker, 2008; Kaufmann et al., 2010; Leaver‐Fay et al., 2011). With statistical and physics‐based calculations, its score function approximates Gibbs free energy (Δ*G*). The ΔΔ*G*, the distinction between the scores of a protein and any of its variants, serves as an estimate of the effects of the substitution, where negative values suggest structural stabilization. ESM scoring is conducted using a locally running instance of ESM‐Scan, in which the protein sequence and a list of amino acid substitutions are provided as input. The default parameters were used: the “esm2_t33_650M_UR50D” model and the “higher accuracy” scoring (a direct implementation of the ‘masked‐marginal’ strategy described in Meier et al., 2021).

Dataset 1 derives from the work of Tsuboyama et al. (2023), which describes a high‐throughput screening of protein variants accompanied by stability measurements. This dataset comprises data of more than 500 amino acid sequences, 32 to 72 residues long, folding as structural motifs or small domains. It includes both natural and Rosetta‐designed de novo sequences from the Protein Data Bank (PDB). ΔΔ*G* values are derived indirectly, by measuring resistance to trypsin/chymotrypsin. Our analysis used a total of 320,000 unique data points from 420 distinct sequences in this dataset to compare measured vs. predicted ΔΔ*G*s.

Dataset 2 contains data on the expression levels and activity of Phosphatase and Tensin homolog (PTEN) variants. Cagiada et al. (2021) compiled this dataset to benchmark their coevolution‐based metric against Rosetta‐generated ΔΔ*G*s and experimental findings from prior studies (Matreyek et al., 2018; Mighell et al., 2018; Suiter et al., 2020). PTEN data derived from studies evaluating cellular growth rate, drug sensitivity, or cellular abundance. The first two metrics are classified as assessments of protein function, whereas the latter is an indicator of protein stability. The PTEN sequence, spanning 403 amino acids, underwent analysis with 5300 individual measurements on function and 7700 on structural integrity.

Furthermore, variant expressibility is binary‐categorized as wild‐type‐like or non‐wild‐type‐like. It is possible to identify threshold values for the ESM and ΔΔ*G* scores that, when compared to this binary classification, accurately predict well‐expressing versus lowly‐expressing variants. This accuracy was measured by mapping Matthews correlation coefficient (φ) trends.

Dataset 3 is a subset drawn from the SKEMPI and ZEMu databases (Dourado & Samuel Coulbourn, 2014; Moal & Fernández‐Recio, 2012), encompassing 66 multimeric proteins with nearly 900 recorded ΔΔ*G*s for amino acid substitutions at protein–protein interfaces, which are long‐studied application targets (Kortemme & Baker, 2002; Smith & Kortemme, 2011; Trepte et al., 2024). Barlow et al. (2018) used this dataset to develop energy‐based methods for variant effect prediction. We calculated substitution ESM scores for individual chains and the multimeric complex. The complex assembly followed the procedure described by Lin et al. (2023), with 25‐residue‐long poly‐G linkers separating chains, a protocol also employed in other studies (Mirdita et al., 2022; Tsaban et al., 2022).

Additionally, ESM scores were computed on a dataset frequently used for in‐house testing in various approaches. Dehouck et al. (2009) curated this dataset to train a multilayer perceptron for predicting ΔΔ*G* using Rosetta‐derived energy terms. Originating from the Protherm database (Gromiha et al., 1999), it includes 2637 experimental measurements of single amino acid substitutions on 129 proteins.

The performance analysis scripts for the three here‐described datasets, plus additional ones, are available at gitlab.tugraz.at/D5B8E35025578B91/esm-scan.

### 2.3 ESM‐scan application to *Ms*LadC

We ran ESM‐Scan to perform a deep mutational scan of *Ms*LadC, whose sequence was obtained from UniProt entry A0A2S5LZS0 and adapted to the expression construct described in Vide et al. (2023). Utilizing the “esm2b_t33_650M_UR50S” model, identified as the optimal‐tradeoff model on our local machines, we scored the variants of *Ms*LadC. We targeted Arginine 218 for further in vitro experiments due to its multifaceted role in allosteric product inhibition and dark‐state conformational stability.

### 2.4 *Ms*LadC mutagenesis and protein production

Guided by ESM‐Scan, we selected single‐substitution *Ms*LadC variants R218K, R218S, R218N, R218A, R218V, and R218D. These variants were generated by one‐step site‐directed mutagenesis, following the protocol described in Liu and Naismith (2008) using pET GB1a *Ms*LadC as a template (Table S1 lists the primers used). The subsequent protein production and purification followed established protocols from Vide et al. (2023). Briefly, plasmids with the respective coding sequences were transformed into *Escherichia coli* BL21 (DE3) cells. Protein expression was induced with 0.1 mM isopropyl‐β‐d‐thiogalactopyranoside at 16°C for 16 h after growth to mid‐log phase in LB medium supplemented with kanamycin (0.03 g/L). The harvested cells were lysed by pulsed sonication, and the soluble fraction was affinity purified using Ni^2+^‐Sepharose (Ni Sepharose 6 Fast Flow, GE Healthcare). The resulting *Ms*LadC variants, featuring N‐terminal solubility (GB1) and purification (6‐His) tags, were concentrated and subjected to size exclusion chromatography. Finally, the purified proteins were flash‐frozen for storage at −80°C.

### 2.5 *Ms*LadC functionality determination

For evaluating *Ms*LadC variant in vitro functionality, we assessed flavin cofactor binding and light state formation via UV–Vis spectroscopy and conducted an in vitro diguanylate cyclase assay to measure enzyme activity. Both analyses followed the established protocols outlined in Vide et al. (2023).

## 3 RESULTS

**FIGURE 1:**
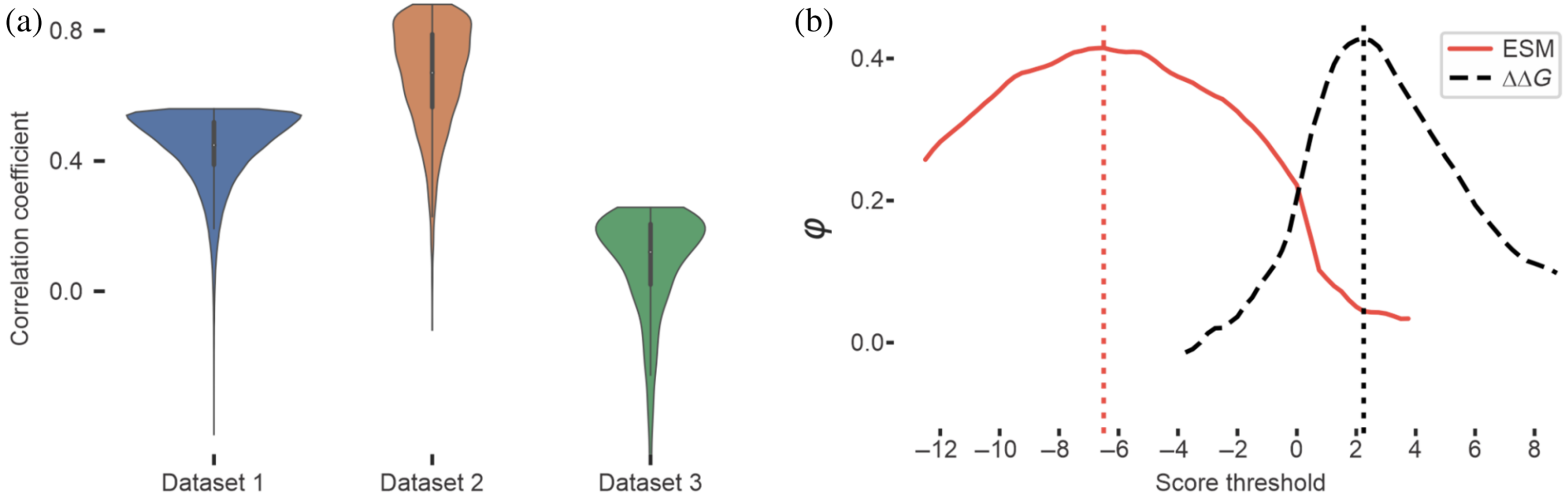
Benchmark correlations and functionality mapping. (a) Correlation and error spread in benchmarked datasets. The distributions depict the probability density function of the normalized mean error for individual data points within each dataset. The means are adjusted to the calculated correlation *R*‐value for enhanced visual representation. (b) Matthews correlation coefficient mappings for PTEN expression data. Mapping of φ values at different ESM and ΔΔ*G* score thresholds for PTEN data from dataset 2. The curve maxima correspond to the best score values to discriminate between well‐expressing and non–well‐expressing variants.

Our results show a correlation between ESM scores and the observed impact of amino acid substitutions in proteins, demonstrating accuracies on par with state‐of‐the‐art methods (Broom et al., 2020). Figure 1a illustrates the prediction accuracies across the main datasets, represented by the relative probability density function. In general, the more skewed each distribution is toward the top, the higher the linearity of the data.

### 3.1 Correlation between experimental, energy‐based calculations and predicted ΔΔ*G* values

Dataset 1 shows an average correlation between the ESM scores and the energy differences caused by the substitutions. ΔΔ*G*s were calculated indirectly through enzymatic digestion resistance, introducing some limitations in accuracy estimates. The overall correlation between ESM scores and ΔΔ*G*s reveals a Pearson R coefficient of 0.44. This correlation value, albeit slightly low, is comparable to those obtained by traditional methods for estimating ΔΔ*G*. A direct comparison on the same dataset using Rosetta calculated ΔΔ*G* values is impractical due to the significant computational requirements necessary for these protocols, highlighting a notable advantage in favor of our tool.

Dataset 2 offers functional and protein expression level measurements for PTEN to compare with Rosetta‐calculated ΔΔ*G*s and coevolutionary scores. ESM scores exhibit a correlation with protein abundance (*R* = 0.48), demonstrating a performance that is comparable to Rosetta ΔΔ*G*s (*R* = 0.49) and coevolutionary score (*R* = 0.48). When considering PTEN functionality, there is a marginal decrease in prediction accuracy for Rosetta and coevolutionary scores (*R* = 0.42 and *R* = 0.46, respectively), while ESM performs significantly better with an *R*‐value of 0.56.

Scanning the ESM and ΔΔ*G* score threshold domains and correlating them with the binary classification for PTEN expression, we could map trends in φ values, as depicted in Figure 1b. The peaks in the curve correspond to threshold values that optimally discriminate between wild‐type‐like‐expressing and lowly expressing variants, as defined in the original study. An ESM score of −6.5 emerges as the most accurate discriminator for this classification criterion. Notably, this analysis also confirms the energy‐based reference ΔΔ*G* values for destabilizing amino acid substitutions, in the range of 2–3 kcal/mol. As with the energy threshold, this ESM score is not a hard limit, but a good guide for educated guesses.

In contrast to the *R*‐value of 0.58 reported by Barlow et al. (2018) for their Rosetta‐based analysis system, ESM‐Scan demonstrates a notable lack of correlation with experimental results for protein–protein interfaces in dataset 3. Even considering the substitutions in the context of multimeric assemblies, the predictive power of ESM does not improve. The recorded correlation coefficient variations are statistically insignificant. It is crucial to note that the model used in this study differs in architecture and size from the ESMFold model described by Lin et al. (2023) despite belonging to the same family. This poor performance is, however, in line with the general weak predictivity of evolutionary conservation methods observed for protein–protein interfaces (Capra & Singh, 2007).

The correlation between ESM scores and the heterogeneous data from Dehouck et al. (2009) reveals only a modest *R*‐value of 0.17. This result, while lower than expected, probably relates to the fact that a variety of protein origins and experimental settings are combined in the dataset. Considering the good correlation with the datasets mentioned above, one should be cautious in overinterpreting the prediction misalignment in this specific case. Furthermore, the model appears to perform at its best when protein functionality and structural stability contribute jointly to the experimental assessment.

### 3.2 Demonstration case— *Ms*LadC

**FIGURE 2:**
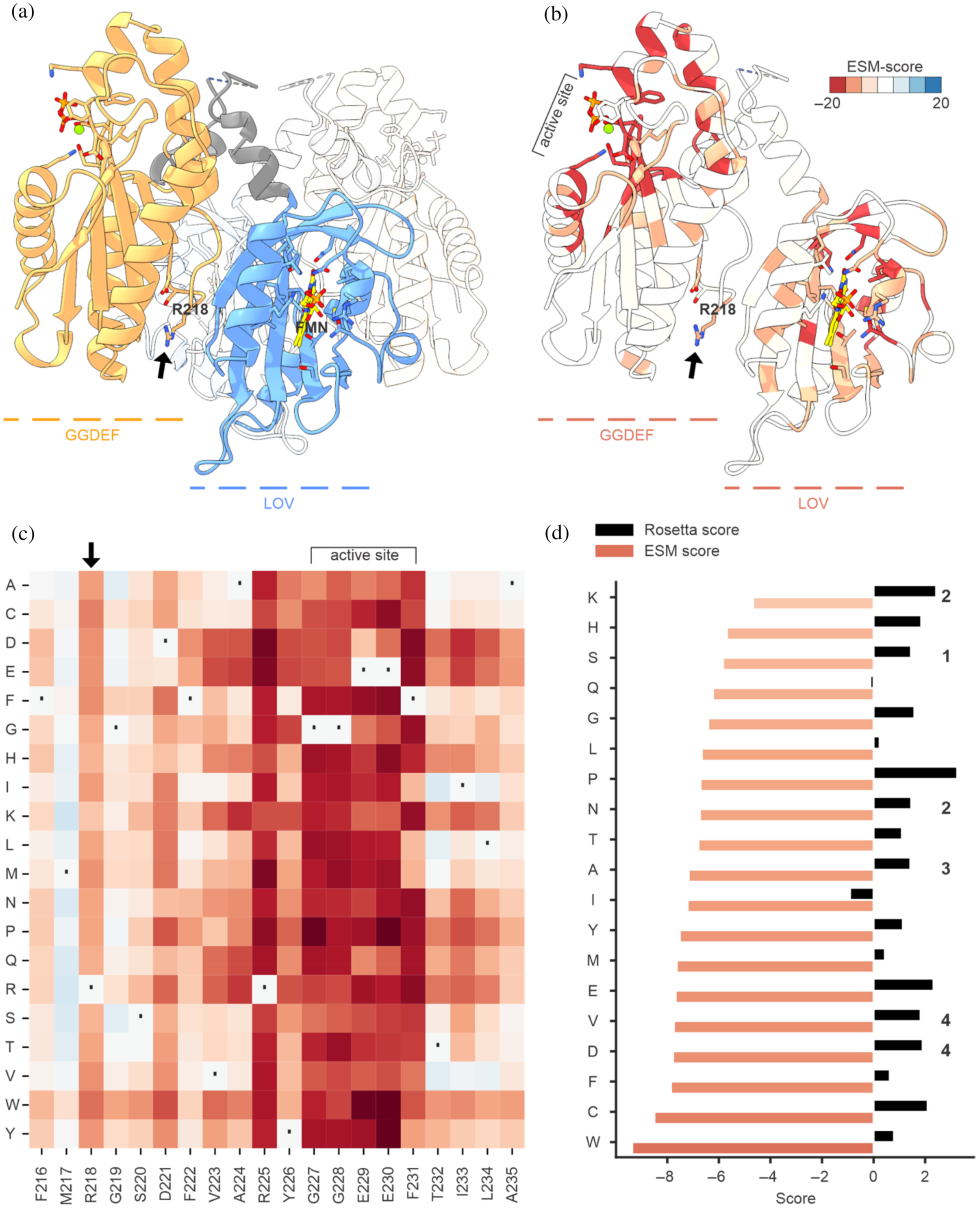
Test case MsLadC. (a, b) Cartoon representation of the MsLadC crystal structure in its dark, inactive state (PDB 8C05). The residues R(218)xxD, lining the inhibitory interface between the LOV and GGDEF domain, are shown as stick models and R218 is indicated by an arrow. The FMN cofactor (yellow), its binding site, and the active site with pyrophosphate (orange) and a Mg^2+^ ion (green) are shown as stick models. In (a), the functionally relevant dimeric state is shown with one LOV domain in blue, the linker helix in gray, the GGDEF domain in orange, and one protomer distinguished in transparency. (b) shows a single protomer colored according to the ESM‐Scan scores heatmap, panel C, averaged at each residue position and binned into seven bins. (c) The ESM‐predicted fitness landscape for the MsLadC deep mutational scan at positions 216–235. The unbinned, continuous color gradient matches the range defined in panel b. The complete heatmap for all residues is shown in Figure S2. A dot indicates wild‐type residues and an arrow points to R218. (d) Comparison of ESM and Rosetta scores for R218 substitutions. Higher ESM values indicate fitter variants, whereas the opposite is true for Rosetta scores. The number next to the columns indicates the experimentally tested variant ranking based on Table S2.

We aimed to generate protein variants of an in‐house model system, *Ms*LadC, that can be expressed and purified, comparable to the wild‐type protein but lacking allosteric product inhibition. We focused on Arginine 218 (Figure 2a,b), located within the characteristic inhibitory site (aa‐sequence motif RxxD), which is involved in the coordination of c‐di‐GMP dimers as part of the diguanylate cyclases' feedback inhibition mechanism (Christen et al., 2006; Schirmer, 2016). Based on the promising results obtained in the benchmarking datasets, we applied ESM‐Scan to *Ms*LadC (Figure 2a–c). Leveraging insights from the ESM‐Scan, we chose to express the *Ms*LadC R218K, R218S, R218N, R218A, R218V, and R218D variants. Despite relatively consistent total expression levels across these variants (Figure S1A), solubility and functionality were significantly affected (Figure S1B and Table S2), highlighting the importance of Arginine 218 in *Ms*LadC, as reflected by the tool's negative scores for substitutions at this position (Figures 2b,c and S2). ESM rankings, purification success, and protein functionality correlate well, with all variants consistently less fit than wild type (Table S2), as anticipated from the diverse roles of R218 and the inhibitory site (see Section 1).

After purification, protein yields for the top‐ranking R218K, R218S, and R218N variants were five times lower than wild type, diminishing to 15 times lower yield for R218A. R218V and R218D even lost the ability to bind the FMN cofactor of the blue‐light sensing LOV domain of *Ms*LadC, resulting in sensory function loss. In the case of the latter two variants, impairment in sensory function was already visible during cell harvest, as the pellets lacked the typical yellow color. While the protein of interest could still be detected on SDS‐PAGE of purified soluble fractions (Figure S1B), the low levels precluded any enzymatic activity assessment. Enzymatic assays for other variants indicated significantly reduced diguanylate cyclase activity compared to the wild type (Table S2). A comparison with the state‐of‐the‐art Rosetta‐calculated energies for potential substitutions at position 218 reveals a lack of correlation with the experimental data (Figure 2d).

Our empirical observations from the *Ms*LadC example align with the observed ESM thresholds calculated for dataset 2 (Figure 1b), where values below −6.5 indicate mainly nonviable substitutions (Figure 2d) and values above it, better‐performing variants.

## 4 CONCLUSIONS

Estimating the effect of single amino acid substitution on protein stability and function is still challenging. Traditionally, efforts have focused on stabilizing protein structures (Broom et al., 2017; Kaufmann et al., 2010). Maximal stability, however, does not always translate to optimal functionality (Gianni et al., 2021; McBride et al., 2022; Shoichet et al., 1995). ESM‐Scan is a broadly applicable and user‐friendly tool designed to predict the functional effects of substitutions based on amino acid sequence. Unlike traditional approaches that focus on the stability of defined structural entities and physical properties, ESM‐Scan likely integrates less well‐defined properties of functionality; furthermore, it also works for proteins lacking validated structures, for example, multistate sensor proteins or even intrinsically disordered systems. Our analysis, comparing predictions to experimental data from deep mutational scanning experiments, offers valuable insights into the potential and limitations of this tool.

ESM‐1v, the original model developed and tested by Meier et al. (2021), demonstrates a certain “understanding” of protein grammar, reporting correlations to experimental results of around 0.5, a value comparable to current state‐of‐the‐art pretrained models (Broom et al., 2020). Our findings point to similar average performances when predicting effects on the stability of monomeric proteins from large datasets. However, ESM‐Scan can yield low‐accuracy predictions, particularly evident if the test data include significant differences in experimental setup or ΔΔ*G* calculation methods. In practical use cases, variable performance should be anticipated, as depicted in Figure S3. Moreover, even though ESM models have been used to model multimeric complexes, predictions at protein–protein interfaces appear unreliable. Whether this is due to model discrepancies, unfitness, or dataset biases, we cannot make a case for this particular use, and we encourage considering alternative tools (Cagiada et al., 2023; Liu et al., 2021; Sieg & Rarey, 2023).

In tasks where spatial features are pre‐eminent, deep learning tools trained on structural information are likely superior predictors, as in the case of protein–protein interactions (Heinzinger et al., 2023; Shu et al., 2023; Wang et al., 2022). Both AlphaFold and ProteinMPNN, a model developed around a message‐passing graph neural network, have shown potential in predicting fitness variations upon residue substitution (Brown et al., 2023; Dauparas et al., 2022; Reeves & Kalyaanamoorthy, 2023). Derivatives, like SaProt and ThermoMPNN, extract the parent models' embeddings and, together with structural information derived from PDB structures, train decoder neural networks to predict fairly accurate ΔΔ*G* values (Dieckhaus et al., 2024; Ouyang‐Zhang et al., 2023; Su et al., 2023; Umerenkov et al., 2023). Such architectures can model conformational free energy surfaces with reasonable approximation, although challenges persist in efficient structure representation and learning (Jing et al., 2020; Ovchinnikov & Huang, 2021). If atomistic data are available, physics‐based tools on the web can provide additional guidance in structural optimization (Goldenzweig et al., 2016; Philipp et al., 2024).

When protein functionality is part of the equation, ESM generally shows state‐of‐the‐art performances. Its inferred values align closely with those provided by co‐evolutionary analysis tools and, in some instances, surpass them. This ability could be confined to naturally occurring proteins or domains included during training; future de novo protein functionality assays might allow testing this hypothesis.

In our example scenario, ESM‐Scan proved helpful in guiding *Ms*LadC substitution variants of a critical amino acid involved in at least two different functionalities: noncompetitive product inhibition and inactive dark‐state assembly formation. Our initial literature‐guided rational approaches failed to yield viable protein variants for thorough in vitro characterization. ESM‐Scan helped us generate functional *Ms*LadC variants and ESM scores correlated strongly with the experimental findings regarding in vitro protein solubility and functionality, while state‐of‐the‐art bio‐physical computational approaches underperformed, highlighting the distinction between stability and more global “functionality”. Furthermore, negative ESM scores generally cluster around functionally important residues and flanking regions (Figure 2b,c), further supporting the notion that scoring relates to a more complex interplay/ensemble of evolution/functionality/stability rather than stability alone as in classical ΔΔ*G*‐estimating approaches. Even if all calculated ESM scores are negative, the best‐ranked substitution likely outperforms the alternatives. Our illustrative set of variants in the MsLadC system confirmed this observation. While further investigation of R(218)xxD‐variants' impact on product inhibition is beyond the scope of this manuscript, the specific activity data presented here underscore the multifaceted role of the inhibitory site, as it strongly influences enzymatic activity also via dynamic coupling with the active site (Christen et al., 2006). While the primary purpose of ESM‐Scan is to guide amino acid substitution choices, examination of the overall scoring (Figure S2) may provide insights, not immediately apparent from MSA‐derived conservation analyses.

Overall, our findings highlight the importance of considering functionality alongside stability in protein engineering efforts. It is important to emphasize that the primary aim of ESM‐Scan is not to enhance or increase protein stability. Other tools, particularly those combined with structural inputs, demonstrably outperform ESM‐Scan in this regard. Rather, ESM extracts information encoded in protein language, potentially revealing nonobvious targets for investigation.

By integrating the ESM model's zero‐shot predictor into an accessible interface we generated ESM‐Scan, an *in silico* deep mutational scanning tool. Geared towards the casual user, it can operate online with minimal setup, computational resource management, or coding expertise. With fast inference times and minimal overhead, the tool is well‐suited for preliminary screenings. Our benchmarking on independent datasets aligns with expected performances. Moreover, we demonstrate the application of ESM‐Scan for conducting an *in silico* deep mutational scan to modulate the functionality of *Ms*LadC. As for any *in silico* prediction, we want to emphasize again that the user must be mindful when interpreting results, testing the model behavior in each case and adjusting threshold values. For advanced users, the flexibility to adapt ESM‐Scan for self‐hosted systems offers possibilities for fine‐tuning to individual needs and potentially superior results in specific instances.

In essence, the ESM model emerges as a robust method for inferring the impact of amino acid substitutions, especially when evolutionary and functional insights are intertwined. Its inferential capabilities present an exciting avenue for *in silico* prediction of protein functionality alterations, potentially reducing the need for resource‐intensive wet laboratory experiments.

## AUTHOR CONTRIBUTIONS

**Massimo G. Totaro:** Conceptualization; investigation; methodology; validation; visualization; writing – original draft; writing – review and editing; software. **Uršula Vide:** Investigation; validation; visualization; writing – review and editing. **Regina Zausinger:** Investigation; writing – review and editing. **Andreas Winkler:** Conceptualization; investigation; funding acquisition; writing – original draft; validation; writing – review and editing; project administration; supervision; resources; formal analysis. **Gustav Oberdorfer:** Conceptualization; investigation; funding acquisition; writing – original draft; methodology; validation; writing – review and editing; formal analysis; project administration; supervision; resources.

## Supporting information


Data S1.
