# Supplementary material for: ESM‐scan—A tool to guide amino acid substitutions
Source: Protein Sci. 2024 Nov 20;33(12):e5221. doi: 10.1002/pro.5221 (PMC11577456; doi:10.1002/pro.5221)
Supplement: Supplementary file 1 — Data S1. [file PRO-33-e5221-s001.docx]

**SUPPLEMENTARY MATERIALS**

**For**

# **ESM-Scan — a tool to guide amino acid substitutions**

#### **Massimo G. Totaro^1^, Uršula Vide^1^, Regina Zausinger^1^, Andreas Winkler^1,2^ and Gustav Oberdorfer^1,2^***

Affiliations:

^1^Institute of Biochemistry, Graz University of Technology, Petersgasse 12/2, 8010 Graz, Austria

^2^BioTechMed Graz, Austria

*correspondence to: [gustav.oberdorfer@tugraz.at](mailto:gustav.oberdorfer@tugraz.at)

**Table of contents**

Figure S1 – S3 (page 2 - 4)

Table S1 – S2 (page 5 -6)


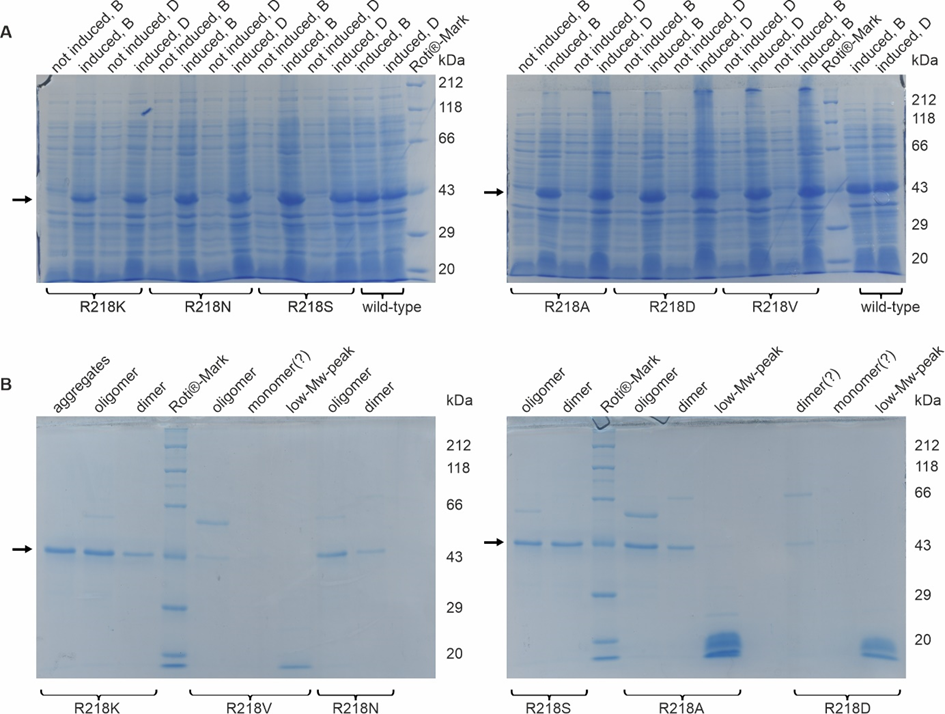
Figure S1: **Analysis of *Ms*LadC-R218 variants overexpression and purification yield**. SDS-PAGE (12.5%) gels in panel **A**) show samples before and after induction with IPTG, both under continuous blue-light illumination (B) and under dark conditions (D). In panel **B**) size-exclusion fractions were analysed, corresponding to aggregates, oligomers, dimers, monomers or other low-molecular-weight molecules.


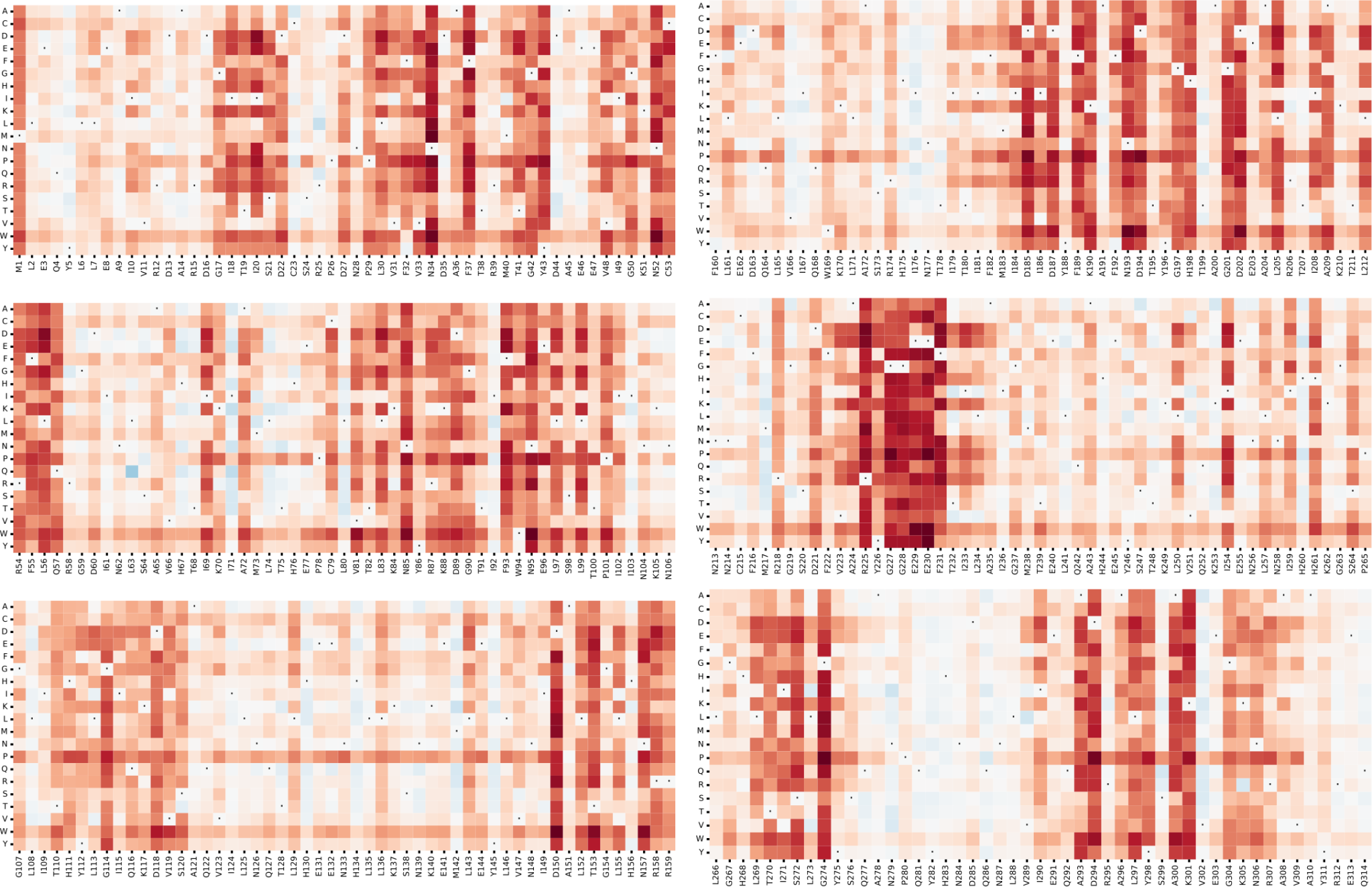

Figure S2: **Deep mutational scanning of *Ms*LadC.** For every residue position of *Ms*LadC, all possible alternative amino acids are scored with the default ESM-Scan parameters. The scores are colour-coded in blue for positive values and red for negative ones, in the range of -20 to 20. The wild-type amino acid scores at zero and is marked by a dot.


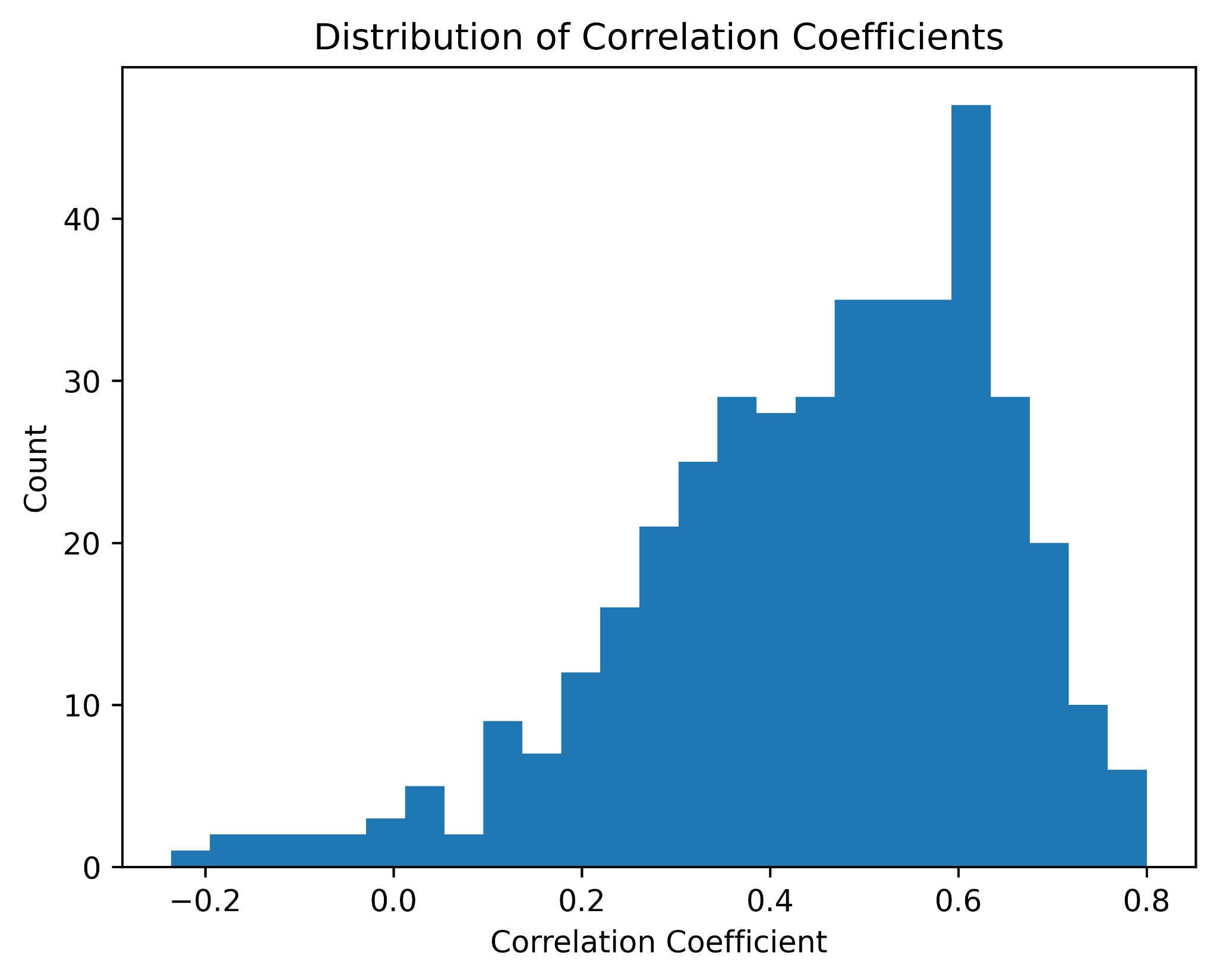

Figure S3: **Distribution of correlations in Dataset 1.** The graph shows the distributions of correlations of all unique sequences in dataset 1. For each of the x sequences the deep mutational scanning yields a correlation value plotted on the x-axis; the sets are unweighted, contributing to one unit on the y-axis independently of the number of experimental data in each.

Table S1: **Primers for site-directed mutagenesis of *Ms*LadC**.

| *Ms*LadC-variant | Forward primer (5’->3’) | Reverse primer (5’->3’) |
| --- | --- | --- |
| R218K | GGCAGCGATTTTGTTGCACGTTATGGTGGTGAAGAATTTACC | CAACAAAATCGCTGCCTTTCATAAAACAATTATTCAGGGTTTTTGCAATGGTAC |
| R218S | GGCAGCGATTTTGTTGCACGTTATGGTGGTGAAGAATTTACC | CAACAAAATCGCTGCCGCTCATAAAACAATTATTCAGGGTTTTTGCAATGGTAC |
| R218N | GGCAGCGATTTTGTTGCACGTTATGGTGGTGAAGAATTTACC | CAACAAAATCGCTGCCGTTCATAAAACAATTATTCAGGGTTTTTGCAATGGTAC |
| R218A | TGTTTTATGGCTGGCAGCGATTTTGTTGCACGTTATGG | GCCAGCCATAAAACAATTATTCAGGGTTTTTGCAATGGTACGCAG |
| R218V | GGCAGCGATTTTGTTGCACGTTATGGTGGTGAAGAATTTACC | CAACAAAATCGCTGCCTACCATAAAACAATTATTCAGGGTTTTTGCAATGGTAC |
| R218D | GGCAGCGATTTTGTTGCACGTTATGGTGGTGAAGAATTTACC | CAACAAAATCGCTGCCGTCCATAAAACAATTATTCAGGGTTTTTGCAATGGTAC |

Table S2: ***Ms*LadC-R218 variants’ overexpression and purification efficiency**. This legend categorises expression efficiency, FMN-bound protein yield and enzymatic activity, determined from the initial velocities of the light-state diguanylate cyclase activities, compared to wild-type behaviour. Symbol Key: ✓ wild-type behaviour; - no functional protein yield.

| *Ms*LadC-variant | Overexpression | Yield compared to wild-type  (based on FMN binding) | Diguanylate cyclase functionality compared to wild-type |
| --- | --- | --- | --- |
| R218K | ✓ | 0.2x | 0.1x |
| R218S | ✓ | 0.2x | 0.5x |
| R218N | ✓ | 0.2x | 0.2x |
| R218A | ✓ | 0.07x | 0.01x |
| R218V | ✓ | - | not measured |
| R218D | ✓ | - | not measured |
